# Removal of Emerging Contaminants (Endocrine Disruptors) Using a Photocatalyst and Detection by High-Performance Liquid Chromatography (HPLC)

**DOI:** 10.3390/ijerph22030334

**Published:** 2025-02-24

**Authors:** Mayra Soares Santos, Amanda Oliveira Mourão, Thuanny Souza Xavier Santos, Mariandry del Valle Rodriguez Rodriguez, Márcia Cristina da Silva Faria, Elton Santos Franco, Núbia Aparecida de Aguilar, Jairo Lisboa Rodrigues

**Affiliations:** Instituto de Ciência, Engenharia e Tecnologia, Universidade Federal dos Vales do Jequitinhonha e Mucuri, Teófilo Otoni 39803-371, MG, Brazil; mayra.soares@ufvjm.edu.br (M.S.S.); amandamourao92@gmail.com (A.O.M.); thuanny.souza.xavier@gmail.com (T.S.X.S.); mariandry.rodriguez.r@gmail.com (M.d.V.R.R.); marcia.faria@ufvjm.edu.br (M.C.d.S.F.); elton.santos@ufvjm.edu.br (E.S.F.); nubia.aparecida@ufvjm.edu.br (N.A.d.A.)

**Keywords:** 17β-estradiol, 17α-ethinyl estradiol, silver arsenate, photodegradation, hormone, nanomaterial

## Abstract

Among several types of emerging contaminants, the endocrine disruptors 17β-estradiol (E2) and 17α-ethinylestradiol (EE2) are particularly notable. These compounds are discharged into sewage systems and subsequently into water bodies, as conventional wastewater treatment processes are unable to effectively eliminate such pollutants. Therefore, the present study aimed to evaluate the possibility of removing the endocrine disruptors 17β-estradiol (E2) and 17α-ethinylestradiol (EE2) from water using the photocatalytic activity of the compound Ag_3_AsO_4_. Silver arsenate was synthesized and characterized, the quantification of the hormones E2 and EE2 was achieved by high-performance liquid chromatography with a fluorescence detector, and a validation process and some preliminary tests were performed on the photodegradation of the hormones using the Ag_3_AsO_4_ catalyst. Validation was performed, and satisfactory results were achieved: r = 0.9987 (E2), r = 0.9984 (EE2), a detection limit of 5.01 (E2) and 0.51 (EE2), a quantification limit of 15.19 (E2) and 1.54 (EE2), coefficients of variation for precision intraday and interday lower than 10.9725% and 11.3393%, respectively, and a recovery of 100.15% (E2) and 100.31% (EE2). In photodegradation studies, Ag_3_AsO_4_ showed different behavior in the presence of light for each hormone. In solution with E2, it reached a removal rate of 35% of the hormone under LED light, acting as a photocatalyst, while with EE2, it reached a removal rate of 96%; both results were obtained after 30 min of exposure to visible light. When this study is compared with other processes and materials, the high efficiency of the Ag_3_AsO_4_ photocatalyst in removing E2 and EE2, persistent emerging contaminants, becomes evident. This advancement has significant implications for wastewater treatment, offering a promising solution that can mitigate environmental impacts caused by endocrine disruptors.

## 1. Introduction

Currently, one of the most serious problems facing the world is environmental pollution in the air, land, and water, with the latter being the most important resource and one that requires special attention [1]. Given the importance of water to the life of all living beings and due to the increase in water needs and consumption due to the continued development of humanity, the generation of wastewater has also increased. At the same time, societies have increased the number of regulations and laws to control water pollution, and these laws require ever higher levels of purification, due to which the continuous improvement of these processes is required. Furthermore, the presence of organic microcontaminants at ultra-trace concentration levels (concentrations below ng L^−1^) is necessitating the creation of specific legislation for their control and treatment.

Organic microcontaminants, which are included among a large number of chemicals that pollute water, comprise organic and inorganic substances from industrial processes, as well as pesticides, medicines, personal care products, and hormones of natural and anthropogenic origin [2,3]. Although microcontaminants are found in low concentrations (concentration levels between ng L^−1^ and μg L^−1^), many of them present serious toxicological problems, because these compounds are biologically active and have a low biodegradability rate [4,5,6].

Endocrine disruptors (EDs) are part of a subclass within emerging microcontaminants. Emerging contaminants can be defined as substances in the environment that are not regulated but have the potential to cause harm to health or the environment, even at low concentrations [7]. According to USEPA [8], an emerging contaminant can be any substance of natural or synthetic origin that can be detected in the environment and can cause harm to biota and humans.

There is still no definitive list of emerging contaminants in the environment. Muñoz [7] proposed a list of some contaminants of great importance in Mexico, considering the volumes used, public health relevance, and toxicological action. In Brazil, Montagnera, Vidala, and Acayaba [9] published a review of what happens in Brazilian aquatic matrices, considering wastewater, surface water, groundwater, and potable water, as well as a discussion of their biological effects and the legislation involving the presence of emerging contaminants, which include personal care products, pharmaceutical compounds, illicit drugs, hormones, pesticides, and other substances considered to be endocrine disruptors.

Naturally, estrogens (estradiol, estrone, and estriol) are mainly produced by developing follicles in the ovaries, the corpus luteum of the placenta, the adrenal cortex, the brain, the testes, the liver, and adipose tissue. These are hormones produced both in men and women but in greater quantities in the latter [10]. The main role of estrogens in the body is to regulate the development, maintenance, and function of the reproductive system in both sexes.

Among these compounds are the natural estrogen 17-β-estradiol (E2) and the synthetic estrogen 17α-ethinylestradiol (EE2), which have high estrogenic activity, even at very low concentrations [11,12]. Depending on the dose and metabolism of the organism exposed to the endocrine disruptor, EE2 is responsible for many adverse effects in aquatic organisms, such as the masculinization of snails, the feminization of fish [13,14], and growth inhibition [14,15], and studies have also shown that exposure to estrogens with levels as low as 1 ng L-1is sufficient to cause the feminization of male trout [16]. EDs also cause disorders in the development of the reproductive system in humans and animals [17].

A possible source of exposure in human beings to high concentrations of estrogen is represented by drugs used in hormone replacement treatments, the regulation of menstrual cycles, or contraceptive methods. EE2, for example, is a synthetic hormone derived from the natural estrogen E2 that is mainly used as an oral contraceptive but is also widely used in hormone replacement treatments and in the cessation of breastfeeding. Estrogens are also used in hormone therapies in domestic animals and livestock. The Registry of Veterinary Specialties (PEV) [18] reports the use of estrogens for therapeutic purposes, such as the use of estradiol for the induction and synchronization of the heat period in cows (Estradiol Benzoate, Bioestrogen, Estrogenic) and estriol for the treatment of urinary incontinence in female dogs (Incurin).

Hormones, steroids, and their metabolites are present in water resources due to the discharge of domestic and industrial effluents that are constantly dumped by humans and animals [19]. The appearance of these compounds is directly related to human development and the techniques adopted in livestock, agriculture, industries, and large urban centers [20,21]. Since effluents from wastewater treatment plants represent one of the main sources of estrogen in the aquatic environment, their degradation through wastewater treatment and their subsequent load on the receiving natural waters is variable, depending on the type of treatment applied, the water retention time, the retention time in sludge, and other operational factors [22,23,24].

Due to its proven presence in the environment and its potential risk to health and wildlife, some initiatives have been developed to regulate the concentration of estrogens in water. The USEPA [25] incorporated estradiol, estriol, estrone, and ethinyl estradiol into its evaluation list of new pollutants, as there is evidence that shows that they have the potential to be endocrine disruptors. Gilbert [26] reported that the European Commission proposed to its member states the establishment of an annual mean concentration limit of ethinyl estradiol of 0.035 ng L^−1^. However, the author stated that there was strong opposition from the pharmaceutical industries to said regulation, because they could guarantee that there was little evidence of harm to the fish population. As these compounds are not completely removed in water treatment, nor in sewage treatment plants, they can reach the aquatic environment.

The concentrations of hormones in the environment are very low, and therefore many analytical methods have been developed to detect and quantify these substances in environmental matrices, such as surface waters [27,28,29,30], drinking water [31,32], sediments [28], and effluent from sewage treatment plants [33,34]. These methods consist of initially preparing the sample through filtration and extraction and subsequently analyzing it using instrumental techniques (gas or liquid chromatography associated with a detector) or non-instrumental techniques (immunoassay or bioassay) [35]. Regarding instrumental techniques, liquid chromatography and gas chromatography have been more widely used for monitoring emerging contaminants in aqueous media [36].

Conventional treatment plants were not designed to remove or degrade emerging contaminants (estrogens), so the effluents from these plants contain significant amounts of estrogens that are dumped and accumulate in the environment. Ternes, Kreckel, and Mueller [37] pioneered the development of methods to remove estrogens from activated sludge from a treatment plant, where estradiol was oxidized to estrone. From this work, several proposals and methods for the retention, degradation or removal of estrogens and others emerging contaminants have emerged [38,39,40,41]. Silva [42] made a summary of some processes for the elimination of estrone, estradiol, estriol, and ethinyl estradiol from water, and among them were biological methods including aerobics, nitrification, anaerobic methods, microalgae, enzymatic treatment systems, and isolated microorganisms; physical methods including adsorption and membrane filtration; and, finally, advanced oxidation processes including photolysis, heterogeneous photocatalysis, strong oxidants, combination of strong oxidants with ultraviolet radiation, photo-Fenton, and sonolysis.

In recent years, the development of photocatalysts activated by visible or UV light has shown promising results in the degradation of organic compounds in water. Among them, photocatalysts using silver (Ag) have been shown to be promising for the photodegradation of organic compounds by irradiation with visible light [43]. Ge et al. [44] synthesized and characterized Ag_3_AsO_4_ using arsenic removed from areas with environmental contamination by mining [45,46], and nanoparticles were used for the photodegradation of Rhodamine B at a neutral pH, achieving the removal of approximately 100% of the dye after 12 min of irradiation. They reused the material up to three times without any significant loss in activity. Bhunia and Jana [47] synthesized and characterized a silver compound with reduced graphene oxide (Ag-rGO) to degrade some colorless endocrine disruptors (phenol, bisphenol A, and atrazine) using UV and visible light; the experiments were carried out by varying the Ag:rGO ratios, and the authors concluded that the photocatalytic efficiency of the material using visible and UV light was satisfactory, dependent on the Ag-rGO ratio and the ability to reuse (recycle) the synthesized photocatalyst up to five times without a significant loss in rate degradation, with reaction times ranging from 20 to 50 min.

This study stands out as an innovative approach, as there are no reports in the literature regarding the use of the Ag_3_AsO_4_ photocatalyst for the degradation of E2 and EE2 compounds. The primary objective was to develop and validate an effective, rapid, and cost-efficient method for determining these compounds in water samples, as well as to investigate their degradation using the Ag_3_AsO_4_ photocatalyst activated by visible light, following its synthesis and characterization, with analysis by HPLC-FLD, to verify the viability of the photocatalyst as an efficient alternative for environmental remediation, offering a promising solution for use in the treatment of emerging contaminants in aquatic environments.

## 2. Materials and Methods

### 2.1. Reagents, Solutions, and Equipment

17β-estradiol (purity ≥ 97%) and 17α-ethinylestradiol (purity ≥ 98%) standard were obtained from Sigma-Aldrich (St. Louis, MO, USA). The solutions were prepared using acetonitrile (for HPLC, grade gradient, purity ≥ 99.9%), obtained from Sigma-Aldrich (St. Louis, MO, USA), and ultrapure water (resistivity 18.2 MΩ cm) obtained using the Barnstead Nanopure system (Thermo Scientific, Waltham, MA, USA). In the synthesis and photodegradation tests, silver nitrate (Plat-Lab, São Paulo, Brazil) and sodium arsenate dibasic heptahydrate (Sigma-Aldrich, St. Louis, MO, USA) were employed. An analytical balance (model FA-2204-BI), a NOVA Instruments heated magnetic stirrer (model NI1109), an Excelsa II FANEM centrifuge (model 206-BL), and a drying and sterilization oven (Solab SL–100) were utilized. Hormone quantification was performed using a high-performance liquid chromatography (HPLC) system equipped with a fluorescence detector (FLD), a quaternary pump, and an autosampler, all from Perkin Elmer Flexar (Waltham, MA, USA).

### 2.2. Synthesis of Ag_3_AsO_4_

Ag_3_AsO_4_ was synthesized following a methodology adapted from Ge et al. [44]. In an Erlenmeyer flask, 0.340 g of AgNO_3_ was dissolved in 15 mL of ultrapure water. In another Erlenmeyer flask, 0.2059 g of Na_2_HAsO_4_ was dissolved in 30 mL of ultrapure water. This solution was added dropwise under constant stirring into the AgNO_3_ solution and stirred for 60 min at 25 °C. The brown precipitate was centrifuged, washed several times with ultrapure water, finally washed with 99% ethyl alcohol, and placed to dry in an oven at 55 °C for 6 h.

### 2.3. Characterization of Ag_3_AsO_4_

The X-ray powder diffraction (XRD) pattern was collected from 20° to 80° 2θ with a Rigaku Geigerflex diffractometer equipped with a graphite diffracted beam monochromator and CuKα radiation (λ = 1.540560 Å). Silicon was used as an external standard. The Shimadzu UV-3600 diffuse reflectance spectrometer (DRS) was used. DRS data were recorded in the range of 200 to 800 nm. The sample was fixed on a sample support (stub), using a TEDPELLA brand double-sided carbon conductive tape. Surface morphology was examined using a HITACHI Scanning Electron Microscope model TM-3000 (Tokyo, Japan) with an OXFORD EDX analyzer model SWIFT ED 3000 (High Wycombe, UK). SEM images were obtained using an accelerating voltage of 15 kV, with magnifications of 1×, 5×, 10×, 100×, and 500×. EDS information was obtained using an accelerating voltage of 15 kV.

### 2.4. Chromatographic Conditions

For the determination of hormones E2 and EE2, we used the chromatographic conditions validated by Carvalho et al. [48]. A high-performance liquid chromatography system with a fluorescence detector was used, equipped with a C18 NTS 18 100 A column (150 mm × 4.6 mm, 5 μm) and an automatic injector with the injection volume programmed to be 20 µL. The mobile phase used in the process was composed of acetonitrile–water (80:20, *v*/*v*). System operation was performed at 29 °C, using a flow rate of 0.8 mL min^−1^; the wavelength used for hormone detection was 280 nm in excitation and 310 nm in emission.

### 2.5. Analytical Validation

The validation parameters applied to the analytical methods included the selectivity, linearity, precision, accuracy, limit of detection (LOD), and limit of quantification (LOQ) [49,50]. Selectivity represents the ability of an analytical method to identify or quantify the target analyte in the presence of other components, either from the sample matrix or potential interferents. Linearity refers to the method’s capacity to produce results directly proportional to the analyte concentration in the sample. Precision assesses the degree of variability among results from independent tests. Accuracy indicates the closeness of individual results obtained by the method to the true or accepted value. The limit of detection is the smallest quantity of the analyte in the sample that can be detected, whereas the limit of quantification is the smallest amount of the analyte that can be quantitatively determined with acceptable levels of precision and accuracy.

Analytical validation for E2 was performed according to the following parameters: linearity (20, 50, 100, 200, 300, 400, and 500 µg L^−1^), intraday precision (200, 400, and 500 µg L^−1^, with three injections of each), intermediate precision (200, 400, and 500 µg L^−1^, with three injections of each), accuracy (100, 200, and 400 µg L^−1^), limit of detection, and limit of quantification. For EE2, the following parameters were studied: linearity (20, 50, 100, 200, and 300 µg L^−1^), intraday precision (50, 100, and 200 µg L^−1^, with three injections each), intermediate precision (50, 100, and 200 µg L^−1^, with three injections of each), accuracy (50, 200, and 300 µg L^−1^), limit of detection, and limit of quantification. All studies were carried out in accordance with INMETRO [49] and ANVISA [50] guidelines.

### 2.6. Photocatalyst Study

The activity of the synthesized compounds was evaluated by removing compounds E2 and EE2 under visible light. An amount of 10 mL of the E2 and EE2 hormone standard with a concentration of 200 μg L^−1^ was dispersed in 10 mg of the photocatalyst and kept in suspension by magnetic stirring in the dark for 30 min at room temperature to obtain the adsorption–desorption equilibrium between the compound and the solution of the corresponding hormone. Then, the suspension was irradiated with a white LED light (λ > 450 nm, 0.55 mW cm^−2^) for 30 min at room temperature. The solution was placed in a falcon tube, where the photocatalyst was separated from the liquid phase by centrifugation and, subsequently, 2 mL of sample was filtered and transferred to a 2 mL vial for subsequent analysis by HPLC-FLD.

## 3. Results and Discussion

### 3.1. Analytical Validation

**Figure 1:**
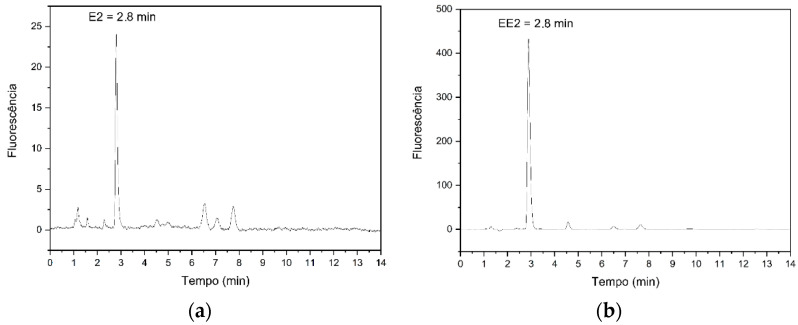
Chromatogram of hormones E2 (**a**) and EE2 (**b**).

The chromatographic conditions used were based on the methodology described by Carvalho et al. [48], with a retention time of 2.8 min (Figure 1) for both compounds, for the analytical validation of endocrine disruptors E2 and EE2, due to their similar structure, differing only by the presence of the ethynyl group linked to position 17.

The evaluated parameters were linearity, precision, accuracy, limit of quantification, and limit of detection.

#### 3.1.1. Linearity

**Figure 2:**
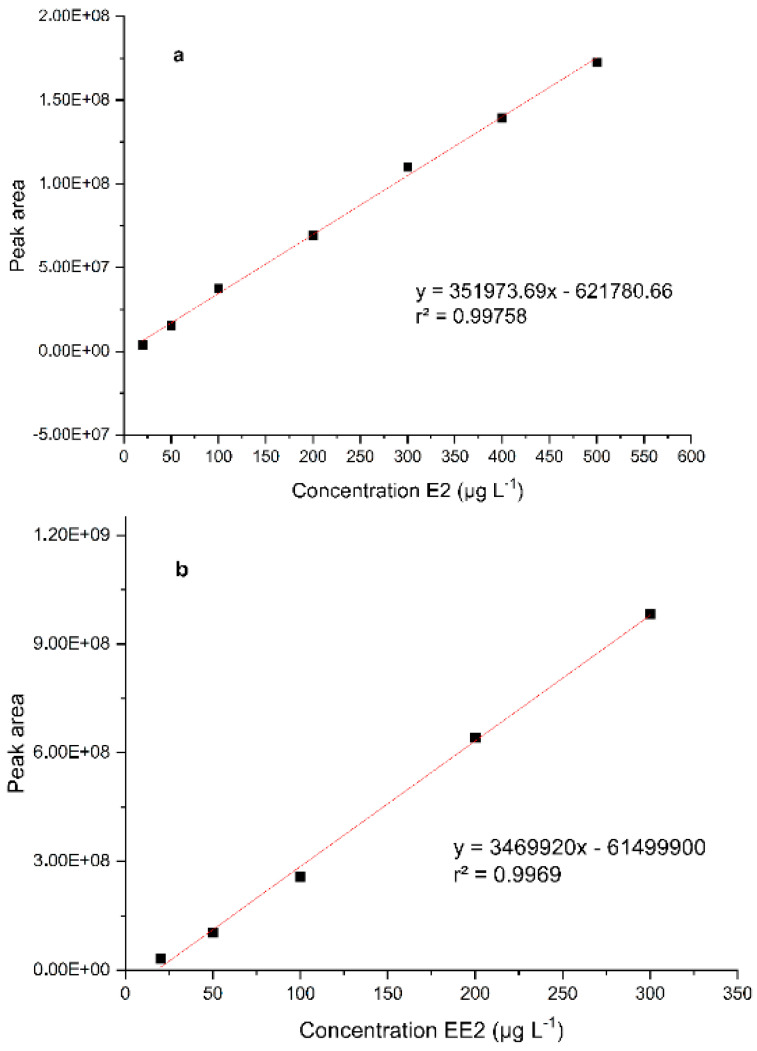
Analytical curve for E2 (**a**) and EE2 (**b**).

The values obtained for the analytical validation of E2 are shown in Table 1 and the analytical curve in Figure 2a, while for EE2, they are shown in Table 1 and Figure 2b, respectively.

The construction of the E2 analytical curve was carried out using concentrations of 20 to 500 µg L^−1^, all triplicate analyses. Through the results, the linear regressions (y = ax + b) were obtained, where y is the peak area and x is the concentration of E2. Similarity was observed between the results in the working range studied and the answers and correlations obtained. A value for the satisfactory correlation coefficient (>0.990) was also achieved, which reaffirms the linearity of this analytical method.

According to ANVISA [50], the linearity of a method shows the ability to obtain analytical responses directly proportional to the concentration of an analyte in a sample. For EE2, the analytical curve (Figure 2) obtained from the studied work range demonstrates a linear relationship between concentrations and areas of peaks obtained in chromatograms. This linearity relationship can be reaffirmed with the observation that the correlation and determination coefficients have values above 0.990.

#### 3.1.2. Precision

Precision studies evaluated the proximity of the results obtained from the same concentration, performed in triplicate. Results both for the same day (intraday) and different days (interday) are expressed with the coefficient of variation (CV) or relative standard deviation (DPR). Table 1 present the results obtained from the precision studies on E2 and EE2.

In Table 1, it is possible to observe that the coefficients of variation in repeatability tests had values below 8.49% for E2 and below 10.97% for EE2, while, for the intermediate accuracy studies, the values did not exceed 11.33% for E2 and 5.59% for EE2. With the limit recommended by INMETRO [49] and AOAC [51] being 15%, the accuracy tests for the quantification of E2 and EE2 show results within the recommended limit.

#### 3.1.3. Accuracy

In the accuracy test, also known as recovery, the agreement is evaluated between individual results in relation to the true value of the certified reference material (MRC).

Table 1 shows the recovery values for the concentrations of 100, 200, and 400 µg L^−1^ for the E2. It is possible to infer that the recovery results had satisfactory values as they showed variations of less than 2% from the real concentration of the MRC.

The data presented in Table 1 were obtained from studies on the EE2 hormone at concentrations of 50, 200, and 300 µg L^−1^. Although the concentration of 50 µg L^−1^ reached a recovery value of 94.96%, all the results were still from 80 to 110% of the recovery recommended by AOAC [51] and INMETRO [49].

#### 3.1.4. Limit of Detection and Limit of Quantification

The limit of detection (LOD) and the limit of quantification (LOQ) are the lowest detectable and quantifiable values of an analyte by a specific method, respectively. The concentrations of these parameters were estimated from white and analytical curves.

The standard white deviation was divided by the angular coefficient of the analytical curve and multiplied by 3.3 for the detection limit and 10 for the quantification limit. In testing for E2 and EE2, the method presented great reliability, being able to detect and quantify the studied analyte even at very low concentrations, as shown in Table 1.

At this exposure, it is possible to state that the method described by Carvalho et al. [48] has a high level of reliability for the quantification of analyses on E2 and EE2, based on the parameters advocated for by INMETRO [49], ANVISA [50], and AOAC [51].

In general, different authors have developed methods to detect estrogens by HPLC analysis. Table 2 shows some works published over the years, the analytical techniques used, the LOQ, and the linear range.

These values are comparable to other validation methods for the determination of the studied hormones. Deki et al. [52] developed a method for quantifying E2, achieving LOD and LOQ results of 9.80 µg L^−1^ and 32.70 µg L^−1^, respectively, while the present work obtained values of 5.01 µg L^−1^ (LOD) and 15.19 µg L^−1^ (LOQ), showing the high effectiveness of the method and the great value of its application for the analysis of low concentrations of contaminants. For EE2, comparing the values obtained in this study with the results presented in Table 2, we can see that the LOQ obtained is well below the others, demonstrating strong potential for use in environmental studies where the concentrations found are low. The developed method was comparable to, or better than, other methods in terms of recovery, reusability, and extraction time.

### 3.2. Ag_3_AsO_4_ Characterization

**Figure 3:**
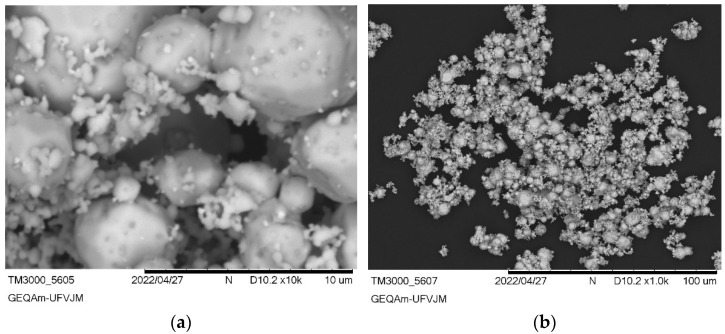
Images of Ag_3_AsO_4_ particles obtained by SEM with magnifications of (**a**) 5× and (**b**) 1×.

The images obtained by SEM of the synthesized Ag_3_AsO_4_ particles, presented in Figure 3, indicate that this material has average particle sizes between 25 and 100 µm and irregular shapes. The percentage breakdown of the elemental chemical composition of the synthesized Ag_3_AsO_4_ obtained by EDS analysis was 12.57% As, 19.66% O, and 67.77% Ag.

**Figure 4:**
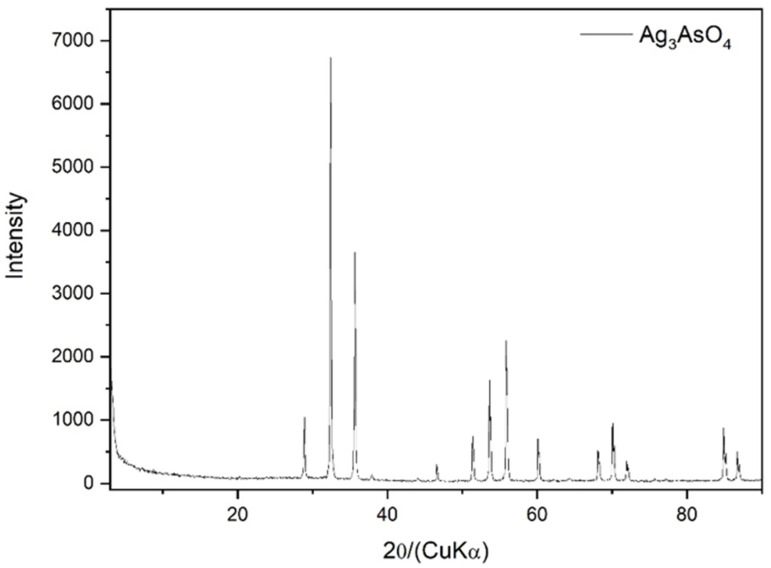
Powder XRD pattern of synthetic Ag_3_AsO_4_.

The identity of the synthesized material was confirmed through XRD analysis (Figure 4). All diffraction peaks could be indexed to the cubic phase of Ag_3_AsO_4_ with JCPDS card number 1-1031, and no other impurity peaks were detected.

**Figure 5:**
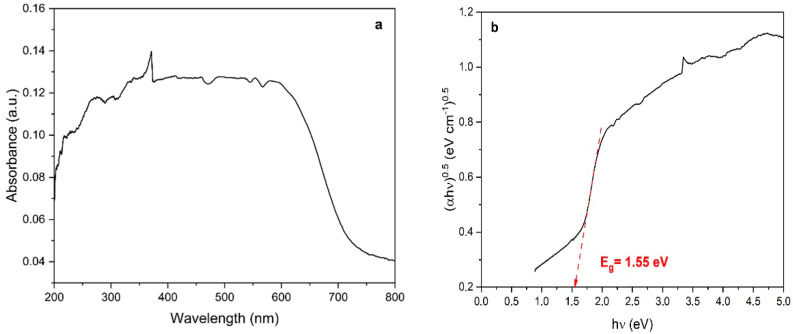
(**a**) UV-Vis DRS spectrum and (**b**) the corresponding Tauc plot for the bandgap determination of Ag_3_AsO_4_.

Figure 5a shows the UV-Vis DRS spectrum of Ag_3_AsO_4_. It can be seen that the synthesized Ag_3_AsO_4_ absorbs visible light, exhibiting the light absorption region from 200 to 700 nm. The optical direct bandgap of the Ag_3_AsO_4_ was calculated using the Tauc equation, αhυ = A(hυ − E_g)^n^, where α, is the linear absorption coefficient, h is Planck’s constant, ν is the light frequency, A is the proportionality constant, Eg is the optical bandgap energy, and n is the transition order; i.e., n = 0.5 for direct transition and n = 2 for indirect transition. According to Tang et al. [56], Ag_3_AsO_4_ is a semiconductor with direct bandgap energy. Observing Figure 5b, we find that 1.55 eV is the optical band gap of Ag_3_AsO_4_. This value is very close to the value reported by Tang et al. [56] and Hott et al. [43].

### 3.3. Hormone Degradation–Adsorption Process Using the Compound Ag_3_AsO_4_

**Figure 6:**
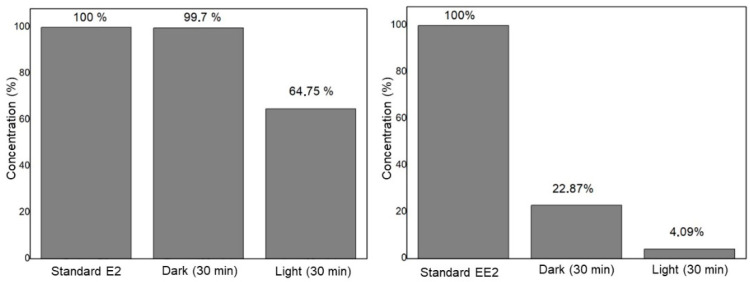
Preliminary study of removal of hormones E2 and EE2 with the compound Ag_3_AsO_4_.

The preliminary study of the activity of Ag_3_AsO_4_ was performed through the degradation of the compounds E2 and EE2 in the dark and under visible light. Figure 6 illustrates the results obtained from the E2 and EE2 removal tests. The first column represents the standard of the compounds, the second column corresponds to the results after 30 min in the dark, and the final column shows the results after 30 min of irradiation.

After the hormone E2 had spent 30 min in the absence of light, there was a removal of 0.3% of the hormone, and after 30 min under magnetic agitation and radiation with LED light, 35% of the hormone E2 were removed. Although it has not yet reached the ideal level of compound degradation, it is possible to infer that silver arsenate has the potential to act as a catalyst in the photodegradation of E2 in visible light, as presented in the works of Hott et al. [43] and Ge et al. [44], which used Ag_3_AsO_4_ in the removal of rhodamine B. Due to this, it is necessary to perform tests, for example, on the kinetics and quantity of the catalyst, to achieve the optimization of the photodegradation process.

For the EE2 hormone, the same test was also carried out for 30 min in the absence of light and 30 min in LED light, both under agitation. In this case, approximately 77% of EE2 was removed in the dark and 18% was removed after exposure to LED light. This shows that Ag_3_AsO_4_ can exhibit two different behaviors when in contact with EE2: first, it acts as an adsorbent material in the absence of light, and then, it acts as a photocatalyst in the presence of light.

The photocatalytic degradation reaction of these organic compounds occurs on the surface of the Ag_3_AsO_4_ photocatalyst, where the absorption of visible light results in the generation of charge carriers (electrons and holes) that initiate the formation of reactive oxygen species. These include hydroxyl radicals (•OH), superoxide (O_2_•^−^), and hydroperoxide (HOO•), which are generated through the interaction between water (H_2_O) and oxygen (O_2_) molecules absorbed on the surface of the semiconductor. These reactive species play a key role in breaking the chemical bonds of the E2 and EE2 compounds, promoting their degradation into smaller products, and, in many cases, complete mineralization. This process results in the conversion of the contaminants into carbon dioxide (CO_2_), water (H_2_O), and other inorganic ions, such as sulfates (SO_4_^2−^) or phosphates (PO_4_^3−^), depending on the conditions of the reaction medium. Therefore, photocatalysis offers an efficient approach to the removal of emerging contaminants, such as E2 and EE2, and their transformation into less toxic and more easily manageable substances in the aquatic environment [57,58,59].

The developed photodegradation method was compared with other studies reporting on the removal of the hormones 17β estradiol and 17α-ethinylestradiol (Table 3). Tian et al. [60] investigated the utilization of the 4,5-seco pathway by the Gram-positive bacterium *Rhodococcus equi* for the degradation of 17β-estradiol (E2). In this study, five genes involved in E2 degradation were identified in *Rhodococcus equi*. Among these, the transformation of E2 mediated by the hsd17b14 gene achieved the highest degradation rate, reaching 63.7% within 30 h. In contrast, Majumder and Gupta [61] employed a photocatalytic approach using 0.47% polythiophene-modified 3.14% Al-doped ZnO for the degradation of the endocrine disruptor E2, achieving approximately 99% removal at a degradation rate of 0.943 h^−1^.

Regarding EE2, Oliveira et al. [62] investigated the use of TiO_2_ and TiO_2_/WO_3_ electrodes for EE2 aqueous solution remediation. Both photocatalysts oxidized EE2 through heterogeneous photocatalysis; however, TiO_2_/WO_3_ exhibited the highest performance for EE2 removal. After 4 h of irradiation, the EE2 degradation rate was 35% with the TiO_2_ electrode, while the TiO_2_/WO_3_ electrode achieved a removal rate of 45%. In contrast, Luo et al. [63] used a novel porous g-C_3_N_4_/reduced graphene oxide/TiO_2_ nanobelt composite for EE2 degradation in the presence of peroxydisulfate and visible light irradiation. This method achieved the complete removal of 6 mg L^−1^ EE2 within 6 min.

This study represents the first investigation into the degradation of E2 and EE2 compounds using the Ag_3_AsO_4_ photocatalyst, demonstrating excellent results within a reduced irradiation time compared to other photocatalysts used for the removal of these endocrine disruptors. Although the removal efficiency for E2 is still below the ideal level, the results indicate significant potential for future process optimization studies. In contrast, EE2 exhibited exceptional removal rates even prior to substantial optimization efforts. These findings underscore the relevance of this study, as they not only highlight the potential for optimization in E2 removal but also demonstrate the effectiveness of the proposed approach for EE2, paving the way for innovative advances in effluent treatment using Ag_3_AsO_4_.

Another significant advantage of this material, compared to others reported in the literature, is its potential for the sustainable reuse of arsenic removed from mining areas. This process not only aids in the mitigation of contamination in mining-affected regions but also contributes to environmental recovery by enabling the treatment of arsenic-contaminated water. Such dual functionality highlights the material’s practical applicability in addressing both pollution control and resource management, as previously noted by Hott et al. [43].

## 4. Conclusions

This study successfully quantified and validated the hormones E2 and EE2 using high-performance liquid chromatography coupled with a fluorescence detector (HPLC-FLD), achieving robust and reliable results. Key performance metrics include correlation coefficients of r = 0.9987 (E2) and r = 0.9984 (EE2), detection limits of 5.01 ng/mL (E2) and 0.51 ng/mL (EE2), quantification limits of 15.19 ng/mL (E2) and 1.54 ng/mL (EE2), intraday and interday precision coefficients of variation below 10.9725% and 11.3393%, respectively, and a recovery of 100.15% (E2) and 100.31% (EE2), confirming the reliability and effectiveness of this analytical methodology.

In photodegradation studies, Ag_3_AsO_4_ exhibited distinct behaviors in the presence of the hormones. For E2, it acted primarily as a photocatalyst, achieving a removal rate of 35% under LED light. In contrast, for EE2, it demonstrated dual functionality as both an adsorbent and a photocatalyst, achieving a significantly higher removal rate of 96%.

These findings highlight the significant potential of Ag_3_AsO_4_ as a photocatalyst for environmental remediation, particularly in addressing hormone contamination in water sources. The reuse of arsenic in the synthesis of silver arsenate further underscores the sustainability and practical relevance of this approach. Future research should focus on optimizing the photodegradation process to enhance removal efficiencies. Additionally, the application of Ag_3_AsO_4_ supported on polymeric matrices for heterogeneous photocatalysis in wastewater treatment should be explored, paving the way for scalable, efficient, and environmentally sustainable solutions to mitigate the impact of endocrine disruptors on natural ecosystems.

## Figures and Tables

**Table 1:** Validation parameters of E2 and EE2.

Analytes	Linear Range (µg L^−1^)	LOD (µg L^−1^)	LOQ (µg L^−1^)	R	Precision (Intraday)		Precision (Interday)		Recovery	
					(µg L^−1^)	%	(µg L^−1^)	%	(µg L^−1^)	%
17-β-estradiol (E2)	20–500	5.01	15.19	0.9987	200	7.3127	200	11.3393	100	100.15
					400	8.4965	400	3.3377	200	98.48
					500	7.4633	500	9.5608	400	99.12
17-α-ethinylestradiol (EE2)	20–300	0.51	1.54	0.9984	50	8.7034	50	5.2371	50	94.96
					100	7.9037	100	5.5929	200	101.23
					200	10.9725	200	4.7280	300	100.31

**Table 2:** Comparison of the method proposed with studies previously reported in the literature.

Reference	Analytes	Linear Range (µg L^−1^)	LOQ (µg L^−1^)	Analytical Technique
[52]	17-β-estradiol (E2)	15–1000	32.10	HPLC-DAD
[53]	17-β-estradiol (E2)	80–1100	80.00	HPLC-UV
	17-α-ethinylestradiol (EE2)	80–1100	80.00	
[54]	17-β-estradiol (E2)	5–500	5.00	HPLC-DAD
	17-α-ethinylestradiol (EE2)	5–500	5.00	
[55]	17-β-estradiol (E2)	300–500	150.00	HPLC-DAD
	17-α-ethinylestradiol (EE2)	600–5000	300.00	
This study	17-β-estradiol (E2)	20–500	15.19	HPLC-FLD
	17-α-ethinylestradiol (EE2)	20–300	1.54	

**Table 3:** Comparison of E2 and EE2 removal rates with those in other studies.

Material	Analyte	Removal Rate	Reference
*Rhodococcus equi*	17 β-estradiol (E2)	63.70%	[60]
Pth/Al-ZnO	17 β-estradiol (E2)	99.00%	[61]
Ag_3_AsO_4_	17β-estradiol (E2)	35.00%	This study
TiO_2_/WO_3_	17α-ethinylestradiol (EE2)	45.00%	[62]
C_3_N_4_/reduced graphene oxide/TiO_2_	17α-ethinylestradiol (EE2)	100%	[63]
Ag_3_AsO_4_	17α-ethinylestradiol (EE2)	96.00%	This study

## Data Availability

The original contributions presented in this study are included in the article. Further inquiries can be directed to the corresponding author.

## References

[1] Salazar M. (2009). Sistemas integrales de tratamiento de aguas residuales, mediante el uso combinado de digestión anaerobia y micro algas. Contactos.

[2] Veronesi M.L.S., Rodriguez M.V.R., Marinho G., Bomfeti C.A., Rocha B.A., Barbosa F., Souza M.C.O., Faria M.C.S., Rodrigues J.L. (2023). Degradation of Praguicide Disulfoton Using Nanocompost and Evaluation of Toxicological Effects. Int. J. Environ. Res. Public Health.

[3] Santos A.P.R., Silva L.Z., Freire B.M., Faria M.C.S., Batista B.L., Rocha B.A., Barbosa F., Rodrigues J.L. (2023). Artisanal Gem Mining in Brazil: A Source of Genotoxicity and Exposure to Toxic Elements. Int. J. Environ. Res. Public Health.

[4] Matos A.R., Faria M.C.S., Freire B.M., Pereira R.M., Batista B.L., Rodrigues J.L. (2022). Determination of 14 trace elements in blood, serum and urine after environmental disaster in the Doce River basin: Relationship between mining waste and metal concentration in the population. J. Trace Elem. Med. Biol..

[5] Mourão A.O., Santos M.S., Costa A.S.V., Silva H.T., Maia L.F.O., Faria M.C.S., Rodriguez M.V.R., Rodrigues J.L. (2023). Assessment of Health Risk and Presence of Metals in Water and Fish Samples from Doce River, Brazil, After Fundão Dam Collapse. Arch. Environ. Contam. Toxicol..

[6] Sun L., Yong W., Chu X., Lin J.M. (2009). Simultaneous determination of 15 steroidal oral contraceptives in water using solid-phase disk extraction followed by high performance liquid chromatography-tandem mass spectrometry. J. Chromatogr. A.

[7] Muñoz C.J.E., Gabriela M., Gerardo B. (2012). Contaminantes emergentes: A aspectos químicos microbiológicos y de salud. Contaminantes Emergentes: Su Importancia, Retos y Perspectivas Sobre la Medición, el Tratamiento y la Reglamentación.

[8] USEPA. United State Environmental Protection Agency (2014). Contaminants of Emerging Concern.

[9] Montagnera C.C., Vidal C., Acayaba R.D. (2017). Contaminantes emergentes em matrizes aquáticas do brasil: Cenário atual e aspectos analíticos, ecotoxicológicos e regulatórios. Quim. Nova..

[10] Hileman B. (1994). Environmental Estrogens Linked to Reproductive Abnormalities, Cancer. Chem. Eng. New.

[11] Jobling S., Sumpter J.P. (2002). Detergent components in sewage effluent are weakly oestrogenic to fish: An in vitro study using rainbow trout (*Oncorhynchus mykiss*) hepatocytes. Aquat. Toxicol..

[12] Ge L., Deng H., Wu F., Deng N. (2008). Microalgae-promoted photodegradation of two endocrine disrupters in aqueous solutions. J. Chem. Technol. Biotechnol..

[13] Desbrow C., Routledge E.J., Brighty G.C., Sumpter J.P., Waldock M. (1998). Identification of Estrogenic Chemicals in STW Effluent. 1. Chemical Fractionation and in Vitro Biological Screening. Environ. Sci. Technol..

[14] Halling-Sorensen B. (2000). Algal toxicity of antibacterial agents used in intensive farming. Chemosphere.

[15] Cleuvers M. (2005). Initial risk assessment for three β-blockers found in the aquatic environment. Chemosphere.

[16] Xuan R., Blassengale A.A., Wang Q. (2008). Degradation of estrogenic hormones in a silt loam soil. J. Agric. Food Chem..

[17] Jiménez-Díaz I., Vela-Soria F., Rodríguez-Gómez R., Zafra-Gómez A., Ballesteros O., Navalón A. (2015). Analytical methods for the assessment of endocrine disrupting chemical exposure during human fetal and lactation stages: A review. Anal. Chim. Acta.

[18] PEV (2014). Prontuario de Especialidades Veterinaria (2014) PLM México. https://www.diccionarioveterinarioplm.com/.

[19] Koh Y.K.K., Chiu T.Y., Boobis A., Cartmell E., Lester J.N., Scrimshaw M.D. (2007). Determination of steroid estrogens in wastewater by high performance liquid chromatographytandem mass spectrometry. J. Chromatogr. A.

[20] Caban M., Lis E., Kumirska J., Stepnowski P. (2015). Determination of pharmaceutical residues in drinking water in Poland using a new SPE-GC-MS(SIM) method based on Speedisk extraction disks and DIMETRIS derivatization. Sci. Total. Environ..

[21] Diamanti-Kandarakis E., Bourguignon J.O., Giudice L.C., Hauser R., Prins G.S., Soto A.M., Zoeller R.T., Gore A.C. (2009). Endocrine-disrupting chemicals: An Endocrine Society 19 scientific statement. Endocr. Rev..

[22] Scognamiglio V., Antonaccia A., Patrolecco L., Lambreva M.D., Litescu S.C., Ghuge S.A., Rea G. (2016). Analytical tools monitoring endocrine disrupting chemicals. TrAC-Trends Anal. Chem..

[23] Silva R.F., Silva G.L., Silva P.T.S., Silva V.L. (2016). Identificação e Quantificação de Contaminantes Emergentes em Estações de Tratamento de Esgoto. Rev. Virtual Química.

[24] Pereira R.O., Postigo C., Alda M.L., Daniel L.A., Barcelo D. (2011). Removal of estrogens through water disinfection processes and formation of by-products. Chemosphere.

[25] USEPA (2012). CCL 3 List Chemical Contaminants [on líne]. United States Environmental Protection Agency. http://water.epa.gov/scitech/drinkingwater/dws/ccl/ccl3.cfm.

[26] Gilbert N. (2012). Drug-Pollution Law All Washed Up. Nature.

[27] Grover D.P., Zhang Z.L., Readman J.W., Zhou J.L. (2009). A comparison of three analytical techniques for the measurement of steroidal estrogens in environmental water samples. Talanta.

[28] Wang L., Ying G.G., Zhao J.L., Liu S., Yang B., Zhou L.J., Tao R., Su H.C. (2011). Assessing estrogenic activity in surface water and sediment of the Liao River system in northeast China using combined chemical and biological tools. Environ. Pollut..

[29] Zhou Y., Zha J., Xu Y., Lei B., Wang Z. (2011). Occurrences of six steroid estrogens from different effluents in Beijing, China. Environ. Monit. Assess..

[30] Zhao L., Deng J., Sun P., Liu J., Ji Y., Nakada N., Qiao Z., Tanaka H., Yang Y. (2018). Nanomaterials for treating emerging contaminants in water by adsorption and photocatalysis: Systematic review and bibliometric analysis. Sci. Total Environ..

[31] Kuster M., Azevedo D.A., López M.J., Aquino F.R., Barceló D. (2009). Analysis of phytoestrogens, progestogens and estrogens in environmental waters from Rio de Janeiro (Brazil). Environ. Int..

[32] Verbinnen R.T., Nunes G.S., Vieirae M. (2010). Determinação de hormônios estrógenos em água potável usando CLAE-DAD. Quimica Nova.

[33] Mohagheghian A., Nabizadeh R., Mesdghinia A., Rastkari N., Mahvi A.H., Alimohammadi M., Yunesian M., Ahmadkhaniha R., Nazmara S. (2014). Distribution of Estrogenic Steroids in Municipal Wastewater Treatment Plants in Tehran, Iran. J. Environ. Health Sci. Eng..

[34] Pessoa G.O., Souza N.C., Vidal C.B., Alves J.A.C., Firmino P.I.M., Nascimento R.F., Santos A.B. (2014). Occurrence and removal of estrogens in Brazilian wastewater treatment plants. Sci. Total Environ..

[35] Fang T.Y., Praveena S.M., Burbure C., Ari A.Z., Ismail S.N.S., Rasdi I. (2016). Analytical techniques for steroid estrogens in water samples—A review. Chemosphere.

[36] Caban M., Migowska N., Stepnowski P., Kwiatkowski M., Kumirska J. (2012). Matrix effects and recovery calculations in analyses of pharmaceuticals based on the determination of β-blockers and β-agonists in environmental samples. J. Chromatogr. A.

[37] Ternes T.A., Kreckel P., Mueller J. (1999). Behaviour and occurrence of estrogens in municipal sewage treatment plants-II. Aerobic batch experiments with activated sludge. Sci. Total Environ..

[38] Andrade T.G., Santos M.S., Maia L.F.O., Aquino T.E.O., Silva L.Z., Silva V.C., Faria M.C.S., Batista B.L., Pedron T., Oliveira L.C.A. (2021). Iron Oxide Nanomaterials for the Removal of Cr(VI) and Pb(II) from Contaminated River After Mariana Mining Disaster. J. Nanosci. Nanotechnol..

[39] Maia L.F.O., Lages G., Ladeira P.C.C., Batista B.L., Faria M.C.S., Oliveira L.C.A., Pereira M.C., Rodrigues J.L. (2021). Development of hollow δ -FeOOH structures for mercury removal from water. Water Pract. Technol..

[40] Santos M.P.O., Santos M.V.N., Matos R.S., Van Der Maas A.S., Faria M.C.S., Batista B.L., Rodrigues J.L., Bomfeti C.A. (2021). Pleurotus strains with remediation potential to remove toxic metals from Doce River contaminated by Samarco dam mine. Int. J. Environ. Sci. Technol..

[41] Hott R.C., Magalhães T.S., Maia L.F.O., Santos K.S.F., Rodrigues G.L., Oliveira L.C.A., Pereira M.C., Faria M.C.S., Carli A.P., Alves C.C.A. (2021). Purification of arsenic-contaminated water using iron molybdate filters and monitoring of their genotoxic, mutagenic, and cytotoxic effects through bioassays. Environ. Sci. Pollut. Res..

[42] Silva L.P. (2021). Desenvolvimento de Métodos Baseados em Cromatografia Líquida Acoplada à Espectrometria de Massas para Determinação de Micotoxinas e Agrotóxicos em Alimentos. 137 f. Tese.

[43] Hott R.C., Andrade T.G., Santos M.S., Lima A.C.F., Faria M.C.S., Bomfeti C.A., Barbosa F., Maia L.F.O., Oliveira L.C., Pereira M.C. (2016). Adsorption of arsenic from water and its recovery as a highly active photocatalyst. Environ. Sci. Pollut. Res..

[44] Ge M., Zhu N., Zhao Y., Li J., Liu L. (2012). Sunlight-Assisted Degradation of Dye Pollutants in Ag_3_PO_4_ Suspension. Ind. Eng. Chem. Res..

[45] Faria M.C.S., Hott R.C., Santos M.J., Santos M.S., Andrade T.G., Bomfeti C.A., Rocha B.A., Barbosa F., Rodrigues J.L. (2023). Arsenic in Mining Areas: Environmental Contamination Routes. Int. J. Environ. Res. Public Health.

[46] Aguilar N.C., Faria M.C.S., Pedron T., Batista B.L., Mesquita J.P., Bomfeti C.A., Rodrigues J. (2019). LIsolation and characterization of bacteria from a brazilian gold mining area with a capacity of arsenic bioaccumulation. Chemosphere.

[47] Bhunia S.K., Jana N.R. (2014). Reduced Graphene Oxide-Silver Nanoparticle Composite as Visible Light Photocatalyst for Degradation of Colorless Endocrine Disruptors. ACS Appl. Mater. Interfaces.

[48] Carvalho R.V., Isecke B.G., Carvalho E., Teran F.J.C. (2017). Photocatalytic oxidation of 17 α-Ethinylestradiol by UVactivated TiO_2_ in batch and continuous-flow reactor. J. Chem. Eng. Mater. Sci..

[49] Instituto Nacional de Metrologia, Normalização e Qualidade Industrial (INMETRO) Orientação sobre Validação de Métodos Analíticos: Documento de Caráter Orientativo (DOQ-CGCRE-008). http://www.inmetro.gov.br/Sidoq/Arquivos/Cgcre/DOQ/DOQ-Cgcre-8_05.pdf.

[50] Agência Nacional de Vigilância Sanitária (2017). Resolução RE No. 899: Guia para Validação de Métodos Analíticos e Bioanalíticos. http://redsang.ial.sp.gov.br/site/docs_leis/vm/vm1.pdf.

[51] AOAC International (2016). Official Methods of Analysis of AOAC International, in Guidelines for Standard Method Performance Requirements (Appendix F). https://www.aoac.org/wp-content/uploads/2019/08/app_f.pdf.

[52] Deki Makkliang F., Pianjing P., Kanatharana P., Thavarungkul P., Thammakhet-Buranachai C. (2023). Natural Luffa cylindrica sponge sorbent for the solid phase extraction of estrone, 17-β-estradiol, and testosterone in aquaculture water. Microchem. J..

[53] Oliveira H.L., Pires B.C., Teixeira L.S., Dinali L.A.F., Simões N.S., Borges W.S., Borges K.B. (2019). Novel restricted access material combined to molecularly imprinted polymer for selective magnetic solid-phase extraction of estrogens from human urine. Microchem. J..

[54] Merib J., Spudeit D.A., Corazza G., Carasek E., Anderson J.L. (2018). Magnetic ionic liquids as versatile extraction phases for the rapid determination of estrogens in human urine by dispersive liquid-liquid microextraction coupled with high-performance liquid chromatography-diode array detection. Anal. Bioanal. Chem..

[55] Almeida C., Nogueira J.M.F. (2006). Determination of steroid sex hormones in water and urine matrices by stir bar sorptive extraction and liquid chromatography with diode array detection. J. Pharm. Biomed. Anal..

[56] Tang J., Liu Y., Li H., Tan Z., Li D. (2013). A novel Ag3AsO4 visible-light-responsive photocatalyst: Facile synthesis and exceptional photocatalytic performance. Chem. Commun..

[57] Heng Z.W., Chong W.C., Pang Y.L., Koo C.H. (2021). An overview of the recent advances of carbon quantum dots/metal oxides in the application of heterogeneous photocatalysis in photodegradation of pollutants towards visible-light and solar energy exploitation. J. Environ. Chem. Eng..

[58] Karim A.V., Krishnan S., Shriwastav A. (2022). An overview of heterogeneous photocatalysis for the degradation of organic compounds: A special emphasis on photocorrosion and reusability. J. Indian Chem. Soc..

[59] Pham T.H., Myung Y., Le Q.V., Kim T.Y. (2022). Visible-light photocatalysis of Ag-doped graphitic carbon nitride for photodegradation of micropollutants in wastewater. Chemosphere.

[60] Tian K., Meng Q., Li S., Chang M., Meng F., Yu Y., Li H., Qiu Q., Shao J., Huo H. (2022). Mechanism of 17β-estradiol degradation by *Rhodococcus equi* via the 4,5-seco pathway and its key genes. Environ. Pollut..

[61] Majumder A., Gupta A.K. (2021). Kinetic modeling of the photocatalytic degradation of 17-β estradiol using polythiophene modified Al-doped ZnO: Influence of operating parameters, interfering ions, and estimation of the degradation pathways. J. Environ. Chem. Eng..

[62] Oliveira H.G., Ferreira L.H., Bertazzoli R., Longo C. (2015). Remediation of 17-α-ethinylestradiol aqueous solution by photocatalysis and electrochemically-assisted photocatalysis using TiO_2_ and TiO_2_/WO_3_ electrodes irradiated by a solar simulator. Water Res..

[63] Luo L., Meng D., He L., Wang X., Xia L., Pan X., Jiang F., Wang H., Dai J. (2022). Photocatalytic activation of peroxydisulfate by a new porous g-C_3_N_4_/reduced graphene oxide/TiO_2_ nanobelts composite for efficient degradation of 17α-ethinylestradiol. Chem. Eng. J..

